# Reduced neural activation during positive social approach is associated with better response to approach avoidance training for social anxiety disorder

**DOI:** 10.1016/j.xjmad.2025.100110

**Published:** 2025-02-07

**Authors:** Christopher Hunt, Morgan M. Caudle, Martin P. Paulus, Murray B. Stein, Charles T. Taylor, Jessica Bomyea

**Affiliations:** a VA San Diego Center of Excellence for Stress and Mental Health, USA; b Department of Psychiatry, University of California, San Diego, USA; c SDSU/UCSD Joint Doctoral Program in Clinical Psychology, USA; d Laureate Institute for Brain Research, USA

**Keywords:** Social anxiety disorder, Approach avoidance training, FMRI, Prediction, Positive affect

## Abstract

**Introduction:**

Accumulating evidence suggests that social anxiety disorder (SAD) is characterized by diminished approach of positive social stimuli. Approach-positive approach-avoidance training (AP-AAT) may reduce this bias, but its results have been mixed. AP-AAT might be more effective for patients with deficits in the neural approach processes AP-AAT targets. Here, we attempted to identify neural areas underlying reduced approach of positive social stimuli in SAD and explore whether activity in such areas predicted response to AP-AAT.

**Method:**

This was a secondary analysis of an AP-AAT clinical trial involving 40 SAD participants and 22 healthy controls (HCs). A social approach-avoidance task was completed during fMRI to identify neural activation differences between SAD and HC subjects when approaching positive social cues. SAD participants were then randomized to AP-AAT (*n* = 18) or sham training (*n* = 22). Treatment response was assessed by changes in positive affect and social connection from pre-to-post treatment.

**Results:**

Compared to HCs, SAD patients exhibited significantly less activation in the left paracentral lobule (PCL), right superior parietal lobule (SPL), and left lingual gyrus (LG) when approaching relative to avoiding positive social cues. Lower activation in the right SPL (b=-7.15, p = .022) and left LG (b=-6.93, p = .007) during social approach versus avoidance predicted greater improvement in positive affect (but not social connection) in the AP-AAT group relative to sham.

**Conclusions:**

Lower neural activation during positive social approach at baseline predicted better AP-AAT response. AP-AAT may be particularly well-suited to SAD patients exhibiting the neural approach deficits that the treatment putatively targets.

**ClinicalTrials.gov Identifier:**

NCT02136212

## 1 Introduction

Though social anxiety disorder (SAD) has traditionally been viewed through the lens of exaggerated negative valence processes (i.e., social fear and avoidance; [2]), accumulating evidence suggests it is also marked by deficits in positive valence systems. SAD patients tend to experience lower levels of positive emotions (e.g., enthusiasm; [23]), are less optimistic about the benefits of social encounters [19], and display fewer positive social behaviors during social encounters (e.g., self-disclosure; [8]). Dysfunction in neurocircuitry underlying positive valence systems, including hypoactivity in neural regions responsible for reward and motivation, is thought to contribute to social impairments in SAD [12], [26], [35]. Novel interventions for SAD are increasingly targeting aspects of the positive valence system given their role in facilitating positive emotional and social outcomes critical to quality of life [1], [5], [9], [32], [36], [37]. These efforts could benefit from neurobiologically-informed treatment targets, including identifying neural substrates that support functioning of positive valence behavior and determining how these brain characteristics predict response to interventions thought to operate via positive valence mechanisms.

One method for assessing positive valence system dysfunction is to measure automatic approach biases for positively-valenced stimuli. In SAD, reduced approach of positive stimuli has been measured using a social adaptation of the approach avoidance task (AAT [21]), which presents images of positive, neutral, or negative social stimuli (happy, neutral, or angry faces) while participants are directed to conduct a simulated approach behavior (arm flexion, i.e., pulling a joystick backward toward themselves) or avoidance behavior (arm extension, i.e., pushing a joystick forward away from themselves). Pulling a stimulus via arm flexion putatively reflects a positive implicit association with that stimulus while pushing a stimulus away via arm extension putatively reflects a negative implicit association [11]. Individuals with SAD consistently display a reduced bias toward positive social stimuli in this paradigm, as observed by quicker reaction times to avoid positive social stimuli (e.g., smiling faces) relative to approaching them [21], [24], [34].

AAT paradigms have also been modified to enhance automatic approach of positive social cues in those with elevated social anxiety. Augmentation of social approach is accomplished by including a disproportionate number of trials in which the target behavior (approach) is paired with the target cue (positive social stimulus), thereby strengthening the implicit association to approach positive social stimuli. Initial studies of this approach-positive approach-avoidance training (AP-AAT) protocol were promising: Relative to sham training, AP-AAT was associated with a greater reduction in bias away from positive social stimuli and greater approach behaviors during a social affiliation task [37] as well as a greater increase in positive mood [32]. While subsequent trials of multi-session AP-AAT training were similarly associated with shifts in approach biases on behavioral [5] and neural [9] levels, they did not find evidence that AP-AAT training improved clinical symptoms to a greater degree than sham training. Such findings underscore the need to better understand the conditions under which AP-AAT training most effectively translates into clinical benefits.

Identifying the neural correlates associated with AP-AAT training offers one way to clarify markers of response, and may also yield information regarding its putative change processes. While the neural basis of AAT task performance in SAD has yet to be investigated, there are several reasons to suspect that it depends at least partially on frontostriatal circuitry. Activity of frontostriatal regions, including the nucleus accumbens, middle cingulate, and precuneus regions, have been linked to greater execution of both implicit and explicit social approach behaviors [30]. SAD patients demonstrate hypoactivity in similar regions during other experimental paradigms involving positive social valence, including reduced activity in the nucleus accumbens in anticipation of social reward [31], the anterior cingulate in response to happy (relative to fearful) faces [33], and the left precuneus during an imaginative social scenario task [29]. Hypoactivity in these regions is thought to contribute to deficient motivation to pursue social rewards and inability to translate positive social reward signals into actual approach decisions [6], [10]. These studies suggest that reduced activation in frontostriatal regions could underlie the bias away from positive social stimuli in SAD. To the extent that symptom reduction in mechanistic interventions like AP-AAT is most marked in individuals experiencing initial deficits in the targeted biobehavioral system [3], [16], [22], neural correlates of approach bias in SAD may also be prognostic of clinical response to AP-AAT. However, no study to our knowledge has investigated the neural correlates of biases on the AAT in any anxiety disorders, let alone investigated the neural correlates of biases away from positive social cues in SAD and how they relate to treatment outcomes from AP-AAT.

The purpose of the present study was two-fold. First, we sought to identify the neural activation differences between individuals with SAD versus healthy controls (HC) during implicit approach of positive social stimuli. In line with reward motivation and approach deficits in SAD being correlated with deficits in frontostriatal circuitry, we hypothesized that SAD patients would show less activation in striatal, ventromedial, and lateral prefrontal regions when approaching positive social stimuli relative to healthy controls. Second, we evaluated whether activation in regions that differed between groups showed associations with clinical response (i.e., changes in positive affect and social connectedness) to an AP-AAT intervention designed to enhance positive approach biases in SAD. For this aim, we conducted a secondary analysis of the primary outcomes (social connectedness, positive affect; NCT02136212) from a previous randomized control trial that compared AP-AAT training plus live social interaction practice to sham training plus live social interaction practice [9]. We hypothesized that hypoactivity in regions linked to positive approach behavior in SAD participants would predict better response to AP-AAT, as reflected by greater increases in positive affect and social connection. This hypothesis is consistent with AP-AAT being more effective for SAD patients exhibiting the neural deficit that the training putatively addresses.

## 2 Methods

### 2.1 Participants

Participants with SAD were recruited from San Diego and the surrounding communities to participate in a computer-based program on positive emotions and behaviors. In addition, healthy control participants were recruited to allow for cross-sectional comparisons of neural responses during the AAT. The final clinical sample consisted of *N* = 40 SAD participants (18 randomized to AP-AAT, 22 to the balanced AAT condition) while the control sample consisted of *N* = 22 participants. Full details on inclusion/exclusion criteria and participant flow can be found in the participant subsection of the Supplemental Materials; the CONSORT diagram from the trial can be found in the Supplemental Materials (Fig S1). Demographic information for both samples can be found in Table 1.

**Table 1:** Demographics and baseline characteristics of the final sample.

	Healthy Controls (*n* = 22)	Approach Positive AAT (*n* = 18)	Balanced AAT (*n* = 22)
**Gender**			
%Male	31.8	22.2	40.9
%Female	68.2	72.2	59.1
%Neither		5.6	0.0
**Race/Ethnicity**			
%Black	0.0	11.1	4.5
% White (non-Hispanic)	31.8	27.8	0.0
% Hispanic	27.73	16.7	40.9
% Asian	27.3	38.9	36.4
% Native American/Alaskan	0.0	0.0	4.5
% Unknown/Declined	0.0	0.0	9.1
% More than one Race	13.6	0.0	9.1
% Other	0.0	5.6	0.0
**Comorbid Diagnosis**			
% MDD	--	22.2	18.2
% GAD	--	16.7	22.7
% PTSD	--	5.6	0.0
% OCD	--	0.0	4.5
**Mean age (SD)**	27.95 (6.97)	22.67 (3.83)	23.86 (5.27)
**Mean Education (SD)**	17.27 (2.85)	15.00 (1.61)	15.14 (1.46)
**Mean PANAS PA (SD)**			
Pretreatment	37.55 (6.51)	24.94 (8.82)	26.82 (7.69)
Posttreatment	--	24.58 (9.42)	25.88 (8.12)
**Mean SCS-R (SD)**			
Pretreatment	108.85 (7.25)	59.28 (12.39)	56.23 (17.40)
Posttreatment	--	65.41 (16.97)	69.81 (19.27)

*Note.* MDD = Major depressive disorder; GAD = Generalized anxiety disorder; PTSD = Posttraumatic stress disorder; OCD = Obsessive compulsive disorder; PANAS PA = Positive and negative affect schedule positive affect subscale; SCS-R = Social Connectedness Scale Revised; SD = Standard deviation.

### 2.2 Procedure

Participants who met inclusion criteria and consented to participate in the study were invited to complete an evaluation session comprised of self-report and behavioral assessments followed by a separate functional magnetic resonance imaging (fMRI) session that was conducted within 1 week of the clinical assessment. Following the fMRI session, the clinical sample was randomized to AP-AAT (treatment arm) versus balanced AAT (control arm) using a simple parallel group 1:1 allocation method via a random number generator. Research personnel and participants were blind to which number corresponded to which condition. Regardless of condition assignment, treatment occurred twice a week for two weeks. Each AAT session was immediately followed by completion of a live social interaction task, which involved a 15-min interaction with a trained confederate in which the participant and confederate alternated responses to a series of questions requiring increasing depth of self-disclosure (for complete details of the clinical trial and task see [9]). Following the final training session, participants completed a post-assessment that was identical to the pre-treatment assessment, including the fMRI scan. All procedures were approved by the University’s Human Research Protections Program (ClinicalTrials.gov Identifier: NCT02136212). The authors assert that all procedures contributing to this work comply with the ethical standards of the relevant national and institutional committees on human experimentation and with the Helsinki Declaration of 1975, as revised in 2008.

### 2.3 Measures: scan task

#### 2.3.1 fMRI AAT assessment

The fMRI AAT assessment was used to obtain indices of neural activity linked to approaching positive social cues. The task involved viewing faces depicting different positive (i.e., happy), negative (i.e., angry), or neutral expressions and using a joystick to pull or push the picture away depending upon color of the border surrounding the picture. All faces were oriented to appear as if they were looking directly at the participant. Participants were instructed that, for each picture, they should pull the joystick if the border was green and push the joystick if the border was blue. Thus, participants were asked to respond only to the color of the border framing each picture, rather than to the content of the image itself. After completing the push or pull movement, participants were instructed to bring the joystick back to the central position, denoted by a small square displayed on the screen. During the task, pulling the joystick made the picture increasingly larger, thereby simulating approach, whereas pushing the joystick made the picture increasingly smaller, simulating avoidance. When the joystick reached approximately 30 degrees in either direction, the picture disappeared, regardless of accuracy. Further details of the AAT paradigm, including length and trial events, can be found in the Procedure section of the Supplemental Materials.

### 2.4 Measures: clinical outcomes

#### 2.4.1 Positive and Negative Affect Schedule, Positive Affect (PANAS-PA; [39]

The PANAS-PA is the 10-item subscale of the PANAS and was used to assess positive affect. Specifically, respondents utilized a 5-point Likert scale to indicate the extent to which they experience a range of different positive emotions (e.g., joy, enthusiasm) over the last week. Internal consistency reliability across timepoints in the current study ranged from very good to excellent (mean Cronbach’s α =.89).

#### 2.4.2 Social Connectedness Scale – Revised (SCS-R; [25])

The SCS-R is a 20-item questionnaire used to assess one’s perception of belonging and interpersonal closeness with others. Respondents utilize a 6-point scale to indicate how much they agree with various statements regarding their current level of belonging/interpersonal connection (e.g., “I am able to connect with other people”). Internal consistency reliability across timepoints for the current sample was excellent (mean Cronbach’s α =.90).

### 2.5 Treatment in SAD group

The AAT treatment program was an adapted version of the AAT assessment that was specifically modified to enhance implicit approach of positive social cues. The AAT treatment protocol was altered in two specific ways relative to the assessment protocol: 1) stimuli consisted of only happy and neutral faces (no angry faces) and 2) the ‘push’ instruction (which simulated avoidance) was replaced with an instruction to move the joystick sideways (equivalent to no approach/avoidance behavior). These changes were made to ensure that participants were not inadvertently trained to approach negative stimuli or avoid positive stimuli.

Within the AAT treatment protocol, there were two conditions: 1) the approach positive condition (AP-AAT; treatment arm), in which most positive pictures were presented with the pull instruction (i.e., 92 % positive, 8 % neutral); and 2) a balanced condition (control arm), in which the pull instruction was evenly paired with each type of picture (50 % positive, 50 % neutral). Because positives images were disproportionately paired with simulated approach (i.e., pull) in the AP-AAT, the AP-AAT should enhance automatic approach of positive social stimuli to a greater degree than the balanced AAT. Further details of AAT treatment procedures can be found in the Procedure section of the Supplemental Materials.

### 2.6 Data analyses: comparison of neural activity between SAD and HC

#### 2.6.1 Imaging analyses

Participants were scanned in a 3-Tesla GE 750 scanner and imaging analyses were conducted using Analysis of Functional Images (AFNI) [13]. For full neuroimaging data acquisition and single-subject analyses details see the fMRI data analysis section in the Supplemental Materials. Voxel-wise percent signal change was the main dependent measure used in general linear models (AFNI 3dLME) comparing activation across groups (SAD versus HC) to contrasts of pull-positive minus push-positive during the baseline AAT task. Two sets of analyses were undertaken. First, an analysis was conducted across the whole brain to examine potential differences in larger-scale frontal regions, including permutation testing (10,000 iterations) within AFNI's updated 3dClustSim [14] to guard against identifying false positive activations (voxel-wise a priori probability of 0.005 with corrected cluster-wise activation probability of 0.05, 71 contiguous voxels [3.5 mm^3^]; see fMRI data analysis section in the Supplemental Materials). Parameter estimates were extracted from the significant clusters and post hoc comparisons were performed to visualize the direction of each effect. Second, in addition to the whole brain analysis, we separately extracted activations for pull-positive minus push-positive within an a priori-defined region-of-interest (ROI) striatum mask (Harvard-Oxford anatomical mask including the caudate, putamen, and nucleus accumbens [NAcc]) to examine group differences in striatal activation in these smaller regions. Extracted values from regions that significantly differentiated SAD from HC across both analyses (whole-brain and ROI) were then used as predictors of positive affect and social connectedness (described below).

### 2.7 Data analyses: relationship between neural response to AAT and positive affect and social connectedness in SAD participants

Linear mixed effect models were used to identify the relationship between baseline neural activity and treatment group in predicting pre- to post-treatment changes in positive affect (PANAS PA) and social connection (SCS-R). Cross-sectional analyses identified three regions of interest (ROIs) that differentiated SAD versus HC at baseline on the AAT assessment (see Results section). Each ROI was tested in a separate model for each of the two outcomes (PANAS-PA, SCS-R), resulting in six total models. Higher ROI values indicate greater neural activity to approaching positive social cues relative to avoiding positive social cues. Each model included a random intercept and tested the fixed effects of the extracted response within the ROI, time, group, each of the three, two-way interactions between these variables, and the three-way Time x Group x ROI interaction. Time was a 2-level within-subjects variable coded such that ‘0’ corresponded to baseline and ‘1’ to posttreatment while group was a 2-level between-subjects variable coded such that ‘0’ corresponded to the AP-AAT condition and ‘1’ corresponded to the balanced AAT condition.

For all models, the main effect of interest was the three-way interaction between the baseline ROI, time, and group, which tested whether the effect of baseline neural activity on changes in positive affect or social connection differed between treatment groups. Each significant three-way interaction was followed up by testing the two-way ROI x Time interaction separately for each treatment group, which helped determine whether baseline activity within a particular neural region was more strongly associated with symptomatic change during the AP-AAT versus the balanced AAT. To account for our testing three ROIs for each outcome, we utilized a Benjamini-Hochberg correction to control for inflated p values resulting from these multiple tests [7]. Behavioral data were analyzed using the Statistical Package for the Social Sciences version 26 (SPSS 26).

## 3 Results

### 3.1 SAD-HC differences in neural activation to approaching positive social cues

**Fig. 1:**
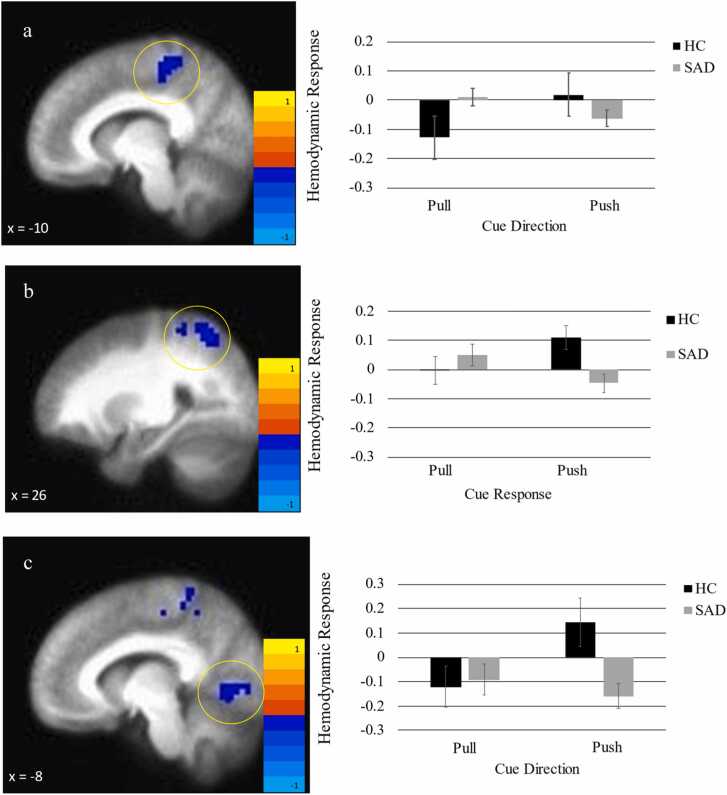
Neural response to pull positive – push positive contrast in SAD versus HC in the a) left paracentral lobule, b) right superior parietal lobule, and c) fusiform/lingual gyrus.

Voxel-wise whole brain analysis of activation during the pull-positive minus push-positive contrast showed a significant effect of group on activation in the left paracentral lobule (PCL) extending into the middle cingulate, right superior parietal lobule (SPL), and the left lingual gyrus (LG) extending into the fusiform gyrus (Table 2, Fig. 1). In all regions, the HC group demonstrated greater BOLD activity to pushing relative to pulling positive faces while the SAD group demonstrated greater activity to pulling relative to pushing positive faces.

**Table 2:** SAD versus HC group differences in pull positive – push positive neural activation.

**Voxels**	**x**	***y***	***z***	***Region***	***BA***	***t***
172	0	−34	57	Left Paracentral Lobule/middle cingulate	5	−3.17
87	27	−55	57	Right Superior Parietal Lobule	7	−3.22
71	−8	−70	−3	Left Lingual/Fusiform Gyrus	18	−3.20

Note. BA = Broadman’s Area

Contrary to expectations, there were no statistically significant group differences in striatal activation in the caudate, *t*(58) = 1.01, *p* = .32, NAcc, *t*(58) = .56, *p* = .58, or putamen, *t*(58) = .87, *p* = .39.

### 3.2 Predicting treatment response from baseline neural activity

#### 3.2.1 Positive Affect

**Fig. 2:**
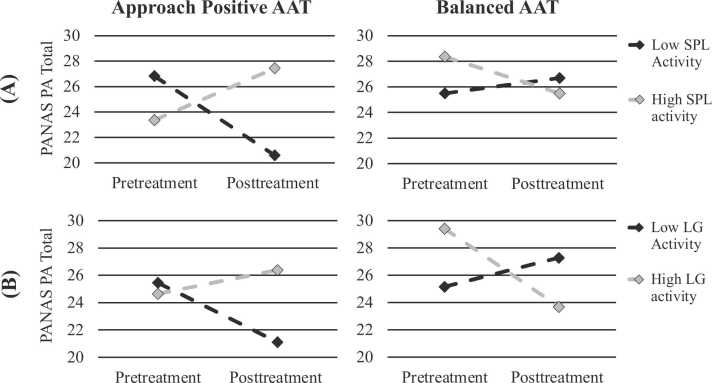
Fitted values illustrating change in PANAS PA scores from pre- to post-treatment across treatment groups based on low (black lines) and high (gray lines) activity within the A) left SPL and B) left LG. Greater neural activity within both regions indicates greater response to the pull condition (approach) relative to the push condition (avoid) toward positively-valanced social cues during the baseline AAT assessment. The low activity lines reflect the estimated PANAS PA change among participants at −1 SD below the sample mean for activity in the region and the high activity line reflects the estimated PANAS PA change among participants at + 1 SD above the sample mean for activity in the region. Approach positive AAT = Approach positive approach-avoidance training; Balanced AAT = Balanced approach-avoidance training; PANAS PA = Positive and negative affective scale positive affect subscale; SPL = superior parietal lobule; LG = lingual gyrus.

Results of mixed effects models for positive affect can be found in Table 3. There was a significant three-way interaction with time and treatment group for activity within both the right SPL, *b*= -7.15, 95 % CI [-13.23, −1.07], *p* = .022, and left LG, *b*= -6.93, 95 %CI [-11.81, −2.05], *p* = .007 (Table 3). In contrast, the three-way interaction between left PCL activity, time, and treatment group was not significant (*p* = .678). Thus, activity during the baseline AAT within the right SPL and left LG, but not the left PCL, predicted changes in positive affect differently in each treatment group. To better understand these effects we next examined the ROI (right SPL, left LG) x time interaction separately for each treatment group. For the AP-AAT group, the two-way interaction with time was in the positive direction for both right SPL activity, *b*= 5.15, 95 % CI [-0.27, 10.58], *p* = .061 and left LG activity, *b*= 3.04, 95 % CI [-1.02, 7.09], *p* = .132, indicating that greater baseline activity to approaching versus avoiding positive social cues within these regions was trending toward predicting a greater increase in positive affect from pre- to post-treatment. In contrast, for the balanced AAT group, the two-way interaction with time was in the negative direction for both left SPL activity, *b*= -2.03, 95 % CI [-4.96, 0.91], *p* = .162 and left LG activity, *b*= -3.93, 95 % CI [-7.01, −0.84], *p* = .016. Thus, greater activity to approaching versus avoiding positive social cues in the left LG and right SPL was associated with a smaller increase in positive affect for the balanced AAT group from pre- to post-treatment, which was statistically significant for the left LG. To illustrate these differences, we constructed a fitted value plot depicting the estimated pre-post treatment change in PANAS PA scores for participants with low (-1SD) versus high (+1 SD) right SPL activity (Fig. 2A) and low versus high left LG activity (Fig. 2B) for each treatment group (balanced AAT and AP-AAT).

**Table 3:** Linear mixed effects models of baseline to posttreatment changes in positive affect.

**Dependent Variable**	**Fixed Effect**	***b***	**95 % CI**	***t***	***p***
*PANAS Total*	Left PCL	3.00	−5.46, 11.48	0.71	.480
	Time	−0.26	−4.10, 3.58	−0.14	.891
	Tx Group	2.02	−3.18, 7.22	0.79	.439
	Left PCL x Tx Group	−4.74	−13.63, 4.15	−1.07	.291
	Left PCL x Time	−0.79	−9.35, 7.78	−0.19	.853
	Time x Tx Group	−0.46	−5.86, 4.94	−0.17	.863
	Left PCL x Time x Tx Group	1.86	−7.15, 10.86	0.42	.678
	Left SPL	1.73	−3.91, 7.38	0.61	.541
	Time	−1.07	−4.68, 2.55	−0.60	.552
	Tx Group	1.83	−3.33, 6.99	0.71	.481
	Left SPL x Tx Group	−3.17	−9.50, 3.17	−1.00	.321
	Left SPL x Time	−5.13	−10.58, 0.32	−1.91	.064
	Time x Tx Group	0.28	−4.79, 5.35	0.11	.912
	Left SPL x Time x Tx Group	7.15	1.07, 13.23	2.39	.022
	Left LG	0.40	−3.79, 4.59	0.19	.858
	Time	−1.30	−4.91, 2.32	−0.73	.498
	Tx Group	2.24	−2.99, 7.47	0.86	.422
	Left LG x Tx Group	−2.53	−7.88, 2.82	−0.95	.374
	Left LG x Time	−3.04	−6.84, 0.77	−1.62	.138
	Time x Tx Group	−0.48	−5.54, 4.57	−0.19	.847
	Left LG x Time x Tx Group	6.93	2.05, 11.81	2.89	.007

Note. Predictors were tested within a model that included a random intercept. Time was a 2-level within-subjects variable that was coded such that 0 = baseline and 1 = post-treatment. Treatment group was a two-level between-subjects variable coded such that 0 corresponded to AP-AAT and 1 corresponded to Balanced AAT. Neural regions were areas whose activations to the push-positive relative to the pull-positive condition of the baseline AAT differed significantly between SAD patients and healthy controls. AP-AAT = Approach-positive approach-avoidance training; PANAS PA = Positive and Negative Affect Scale Positive Affect subscale; PCL = Paracentral lobule; SPL = Superior parietal lobule; LG = Lingual gyrus; Tx = Treatment; CI = Confidence interval.

#### 3.2.2 Social connection

In contrast to PANAS PA models, none of the three-way interactions between time, treatment group, and baseline neural activity were significantly associated with SCS-R scores (*p*s > .210; Table 3). Thus, the ability of the left PCL, right SPL, or left LG to predict pre-post changes in social connection did not differ between treatment groups. Complete results of mixed effects models for social connection can be found in Table S1 of the supplemental materials.

## 4 Discussion

The purpose of this study was to elucidate the neural underpinnings of biases away from positive social stimuli in SAD and to determine if these neural markers were predictive of response to an approach-positive approach-avoidance training (AP-AAT) intervention designed to mitigate this bias. Contrary to our hypothesis, neural activation differences between SAD patients and HC were not present in frontostriatal areas but were predominantly observed in the parietal and occipital regions, notably the left paracentral lobule, right superior parietal lobule, and left lingual gyrus. Decreased activity in the right superior parietal lobule and left lingual gyrus during positive social approach was associated with enhanced positive affect following AP-AAT relative to sham training. These outcomes challenge our initial hypotheses, suggesting that regions outside of frontostriatal circuitry may contribute to approach biases in SAD and may serve as indicators of therapeutic efficacy in approach-avoidance training interventions such as AP-AAT.

The absence of differences between SAD patients and HCs in frontostriatal areas during the approach of positive stimuli suggest that approach-related dysfunction is SAD may stem from different neural circuits than have been linked to approach-avoidance deficits in other disorders [6], [10], [15], [41] – specifically visual processing or sensorimotor areas. While unexpected, the role of these areas in approach avoidance tasks is not without precedent. For instance, increased activation of the fusiform and lingual gyrus has been observed during the approach of positive valence [4], [15] and the parietal cortex has been linked to differences in valence during the AAT [4]. Future research is needed to determine whether differences in how SAD patients view and respond to positive social stimuli contribute to neural activity during approach processing. Individuals with SAD commonly display an aversive reaction to positive social feedback or events [40], perhaps contributing to observed parietal and fusiform activity that has been linked to the anticipation of threatening cues [18], [27], [28] and to SAD [17], [42]. Perception of positive social stimuli as threatening could also create behavioral conflict when SAD participants are directed to approach them. Heightened neural activity during conflict within approach-avoidance tasks (i.e., incongruent trials) has been observed in similar regions as those linked to SAD-related approach deficits in the current study (e.g., parietal regions: [4], [10], [38]; middle cingulate: [4]; lingual gyrus: [10]), including in a study of anxiety disorder patients [10].

Greater activity in two of the three regions linked to positive social approach in SAD (right superior parietal lobule, left lingual gyrus) predicted greater increases in positive affect in response to AP-AAT (relative to the balanced AAT). This finding is broadly consistent with an experimental therapeutics model, whereby treatments are best suited for those exhibiting greater dysfunction in a specific pathological target that is mechanistically linked to clinical outcomes [20]. In our study, deficient activity in the right superior parietal lobule and left lingual gyrus may have afforded greater opportunity for correcting biases away from positive social stimuli, allowing for greater clinical improvement. If replicated, these findings could be used to develop treatments that are tailored to these specific neural sensitivities which could ultimately help optimize therapeutic outcomes for SAD patients.

The present findings should be considered in the context of several limitations. First, our small sample size limits our ability to detect smaller size effects; thus, the present results should be replicated in a larger sample to determine whether other neural areas might also be linked to positive approach biases in SAD or are predictive of response to AP-AAT. Second, our control condition included some active components that may have contributed to similar changes in social connection (e.g., live social interaction practice), making it difficult to identify predictors of social connection change that are specific to AP-AAT. Finally, there are several differences between our task design and those utilized in prior fMRI studies of approach-avoidance that preclude direct comparison of findings. For instance, our study examined implicit rather than explicit cue processing and contrasted approach versus avoidance of positive stimuli rather than congruent versus incongruent trials. The extent to which these discrepancies contributed to the lack of differences in frontostriatal activity identified in past investigations will need to be examined in future work. Despite these limitations, the current findings are useful for generating hypotheses about the neural correlates of positive approach biases in SAD and their relation to an intervention like AP-AAT that is designed to eliminate such biases.

In summary, this study sought to identify the neural correlates of positive approach bias in SAD and examine whether such activity predicted response to an approach-positive approach-avoidance training intervention designed to ameliorate this bias. Unexpectedly, results indicated that SAD participants showed greater activation than healthy controls in visual and sensorimotor processing regions when approaching (relative to avoiding) positive social stimuli and that a subset of these regions predicted better response to the AP-AAT intervention. Methodological limitations, particularly a small sample size, necessitate replication; however, our findings tentatively imply that AP-AAT may be most beneficial for SAD patients whose neural response to approaching positive stimuli is most aberrant from that of psychologically healthy individuals. Future work may build upon these findings by testing the effectiveness of prospectively matching SAD patients to AAT based on their baseline neural profile, which may help further clarify the mechanisms underlying approach-avoidance training interventions for positive approach biases in SAD.

## Funding statement

This study was funded through a grant from the 10.13039/100000025National Institute of Mental Health awarded to CT (R00MH090243). Individual authors contributions were supported by grant funding from VA CSR&D (1IK2CX002640, CH; IK2CX002640, JB), VA RR&D (RX003793, JB), the National Institute of Mental Health (R61MH127005, JB), The 10.13039/100001380William K. Warren Foundation (MP), the National Institute of General Medical Sciences Center (Grant 2 P2GM123120, MP), the 10.13039/100000026National Institute on Drug Abuse (U01DA50989, MP).

## Declaration of Competing Interest

MP advises Spring Care, Inc., receives royalties from an article on methamphetamine in UpToDate, and has a compensated consulting agreement with Boehringer Ingelheim International GmbH. All other authors declare no possible competing interests.
