# Supplementary material for: Reduced neural activation during positive social approach is associated with better response to approach avoidance training for social anxiety disorder
Source: J Mood Anxiety Disord. 2025 Feb 7;10:100110. doi: 10.1016/j.xjmad.2025.100110 (PMC12244139; doi:10.1016/j.xjmad.2025.100110)
Supplement: Supplementary file 1 — Supplementary material [file mmc1.docx]

**Reduced Neural Activation During Positive Social Approach is Associated with Better Response to Approach Avoidance Training for Social Anxiety Disorder**

Christopher Hunt^a^, Morgan Caudle^b^, Martin P. Paulus^c^, Murray B. Stein^d^, Charles T. Taylor^d^**,** Jessica Bomyea^a,d^

^a^VA San Diego Center of Excellence for Stress and Mental Health

^b^SDSU/UCSD Joint Doctoral Program in Clinical Psychology

^c^Laureate Institute for Brain Research

^d^University of California, San Diego

***Supplemental Materials***

**Methods**

**Participants**

Participants were required to be between the ages of 18 to 55, to meet criteria for a principal diagnosis of SAD as assessed via the Structured Clinical Interview for the Diagnostic and Statistical Manual (4th ed.; DSM–IV (APA, 2000)) Axis 1 Disorders, SAD module (SCID-I (First, 2004)), and to score 50 or higher on the Liebowitz Social Anxiety Scale (LSAS (Liebowitz, 1987)). Exclusion criteria entailed: (1) active suicidal ideation with intent or plan; (2) moderate to severe alcohol or marijuana use disorder within the past year; (3) all other mild substance use disorders within the past year; (4) bipolar I disorder; (5) psychotic disorders; (6) moderate to severe traumatic brain injury with evidence of neurological deficits or neurological disorders; (7) severe or unstable medical conditions that might leave the individual negatively impacted by participation in the study; (8) inability to speak or understand English; (concurrent psychotropic medication that could affect brain function (e.g., SSRIs, benzodiazepines); and (9) characteristics that would compromise safety to complete an MRI scan (e.g., metal devices in body).

Of the 79 SAD participants who were assessed for study eligibility, 57 met eligibility criteria, of which 12 dropped or were withdrawn from the study prior to randomization while five were excluded from analysis due to issues with fMRI data collection (see Supplemental Materials, fMRI Data Analysis, for more details). Of note, five subjects (*n* = 4 balanced AAT, *n* = 1 AP-AAT) did not have post-treatment symptom data but were nonetheless included in analyses, consistent with an intent-to-treat design. The healthy control sample had the same inclusion/exclusion criteria as the clinical sample with the additional specifier that they did not meet for any assessed DSM-5 mental health diagnosis and scored below 20 on the Liebowitz Social Anxiety Scale (Liebowitz, 1987).

**Procedure**

***AAT Assessment*.** Participants completed two 96 trial runs for a total of 192 trials [8 Pictures x 3 Picture Type (happy, neutral, angry) x 2 Gender (male, female) x 2 Border Color (green, blue) x 2 Repetition]. Trials were presented in a pseudorandom order that was consistent across participants. The task began with a fixation cross appearing for 12 seconds. Following the fixation was the appearance of a medium-sized picture (400 x 400 pixels) in the center of the screen. The computer program logged the position of the joystick and the images were fluidly resized as the joystick was moved from the central position as follows: The pictures became increasingly larger if the participant pulled the joystick, simulating approach, and increasingly smaller if the participant pushed the joystick, simulating avoidance. Moving the joystick to the right or left did not change the size of the picture. When the joystick reached approximately a 30° position in either direction, the picture disappeared, regardless of whether the participant responded correctly. Interstimulus intervals (ISIs) were 2500, 3000, or 3500 milliseconds; ISI order was pseudorandomized with the same proportion of ISIs across each run. Each 96-trial run was comprised of four blocks of 24 trials separated by 12 second fixation breaks, including at the start and end of the run (totaling 5 fixation breaks). The face stimuli were taken from the Karolinska Directed Emotional Faces stimulus set (KDEF (Goeleven, De Raedt, Leyman, & Verschuere, 2008)) and was comprised of two sets (A and B) 16 actors (8 female, 8 male) displaying a range of emotional expressions (happy, neutral, and angry faces). Consistent with previous research (Najmi, Kuckertz, & Amir, 2010; Taylor & Amir, 2012), colored frames surrounding each picture were used to guide participants’ direction of movement.

Before completing the task in the scanner, participants first completed a practice version of the task (12 trials). The fMRI AAT was administered at both pre- and post-treatment; however, only the pretreatment assessment was examined for the current study.

**Treatment in SAD group**

The AP-AAT intervention was an adapted version of the AAT assessment and was specifically modified to enhance automatic action tendencies toward approaching positive social cues. As with the original AAT task, participants were directed to make specific movements with a joystick in response to the border color of a facial image. However, in the AP-AAT protocol, the ‘push’ instruction (which simulated avoidance) was replaced with an instruction to move the joystick sideway (equivalent to no behavior), which resulted in no change to the size of the image. More specifically, participants were instructed to pull the joystick if the border was green and move the joystick to the right if the border was beige. Push trials were eliminated during the AP-AAT protocol for two main reasons: First, the primary aim of the treatment was to manipulate approach tendencies rather than manipulate *both* approach and avoidance tendencies, which could be accomplished by the elimination of push trials that simulated avoidance. Second, instructing participants to make the push command would result in them avoiding neutral cues, which could dilute the ability of the manipulation to alter automatic tendencies in the direction of approach. For similar reasons, the AP-AAT protocol only utilized happy and neutral faces (no angry faces) since altering tendencies in a way that motivated more automatic approach of negatively valenced stimuli ran contrary to the purpose of the treatment (i.e., increase approach of positive social cues). As with the baseline AAT assessment, participants completed 12 practice trials with non-trained stimuli to become familiar with the instructions before completing the AAT session.

***Approach Positive AAT.*** In the approach positive AAT (AP-AAT) condition, the majority of positive pictures (92%) during were presented with the pull instruction versus 8% that were presented with the sideways instruction. These same contingencies were reversed for the neutral pictures (8% in the pull format and 92% in the sideways format). Thus, positive pictures in the AP-AAT were disproportionately paired with simulated approach (i.e., pull), which was done to alter automatic tendences in the direction of approaching positive social cues. The total number of pull and sideways trials remained evenly split (50% of trials each). The experimental phase comprised 384 trials with a short break in between (i.e., two runs of 192 trials each): 8 facial images x 2 emotions (positive, neutral) x 2 border colors (green, beige) x 12 Repetitions. The training phase was approximately 15 minutes in duration.

***Balanced AAT.*** The balanced approach condition was identical to the AP-AAT except that positive and neutral pictures were paired with an equivalent proportion of pull and sideways directions (50%) each. Thus, participants in the balanced AAT were required to make an *equal number* of approach (pull) and sideways (move to the right) movements for positive social cues and equal number of approach and sideways movements for neutral stimuli. Since positive social cues were not disproportionately paired with approach behavior in the balanced AAT, the balanced AAT should theoretically not result in any net change in automatic approach tendencies toward positive social cues.

**fMRI Data Analysis**

Participants were scanned using an 8-channel head array coil. Each scanning session included a three-plane scout scan, a sagittally acquired spoiled gradient recalled (SPGR) sequence for acquiring T1-weighted images (172 slices; thickness=1mm; TI=450ms, TR=8ms, TE=3ms; matrix=192 x 256; FOV=256 cm; flip angle=12ﹾ; sagittal plane) and T2*-weighted axially acquired echo-planar imaging (EPI) scans to measure blood oxygen level dependent (BOLD) signals (slice thickness=3mm; slice spacing=1mm; TR=1.5s, TE=32ms, flip angle=80ﹾ; matrix=64 x 64, FOV=240mm). The task was administered over two runs.

Imaging analyses were conducted using Analysis of Functional Images (AFNI). Standard preprocessing steps were used with the afni.proc.py tool including removal of outlying acquisitions, despiking, slice time correction, co-registration of anatomical and functional scans, spatial smoothing (6-mm half maximum smoothing kernel) and warping to standardized MNI space. Prior to conducting analyses, behavioral and fMRI data were inspected for each participant (see below).

Regressors from the fMRI task were shifted by a hemodynamic waveform (AFNI:waver) and individual preprocessed EPI data were entered into a general linear model. Regressors of no interest included motion parameters and baseline. Regressors of interest were generated based on coding trials per the two directional conditions (push, pull) and three valence types (angry, positive, neutral). Prior to conducting analyses, behavioral and fMRI data were inspected for each participant and fMRI AAT data from *n* = 5 SAD group participants and *n =* 4 HC group participants were removed due to poor quality based on visual inspection of alignment and motion.

Figure S1. CONSORT Diagram of SAD sample in the current study.


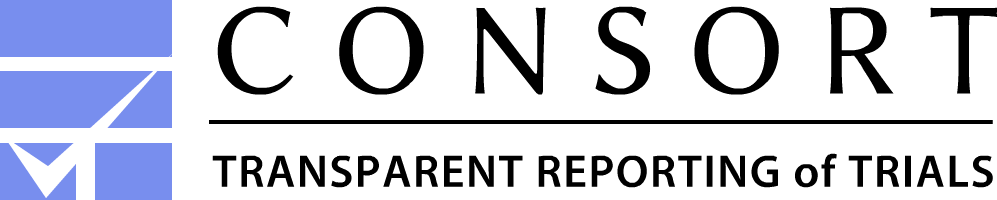


**Assessed for eligibility (n= 79)**

## Enrollment

**Excluded (n= 22)**

♦ Not meeting inclusion criteria (n=21)

♦ Declined to participate (n=1)

**Enrolled (n=57)**

Dropped Prior to Randomization (n = 12)

Dropped due to time commitment (n=3)

Dropped due to claustrophobia/MRI issues (n=3)

Withdrawn due to concurrent treatment (n = 1) Began medication (n = 1) ¨ Lost to contact (n = 3) Uncomfortable with study procedures (n = 1)

**Randomized (n=45)**

## Allocation - parallel 1:1

**Approach Positive AAT (n=21)**

♦ Dropped before intervention (n=1)

- Lost to contact (n=1)

♦ Dropped during intervention (n=0)

**Completed post (n=20)**

**Balanced AAT (n=24)**

♦ Dropped before intervention (n=4)

- Time commitment (n=1)

- Lost to contact (n=3)

♦ Dropped during intervention (n=2)

- Lost to contact (n=2)

**Completed post (n=18)**

## Analysis

**Analysed (n= 22)**

♦ fMRI data removed (n = 2)

- poor quality based on inspection of alignment and motion (n = 2)

**Analysed (n= 18)**

♦ fMRI data removed (n = 3)

- poor quality based on inspection of alignment and motion (n = 3)

Note. Approach positive AAT = Approach-positive approach-avoidance training; balanced AAT = Balanced approach-avoidance trainig; fMRI = Functional magnetic resonance imaging.

**Table 1S.** Linear mixed effects models of baseline to posttreatment changes in social connection

| **Dependent Variable** | **Fixed Effect** | ***b*** | ***95% CI*** | ***t*** | ***p*** |
| --- | --- | --- | --- | --- | --- |
| *SCS-R Total* | Left PCL | 6.06 | -10.53, 22.65 | 0.73 | .467 |
|  | Time | -5.80 | -0.45, 11.65 | 2.02 | .052 |
|  | Tx Group | 3.92 | -14.09, 6.26 | -0.77 | .443 |
|  | Left PCL x Tx Group | -2.11 | -19.51, 15.30 | -0.24 | .809 |
|  | Left PCL x Time | 0.52 | -12.57, 13.61 | 0.08 | .936 |
|  | Time x Tx Group | 6.95 | -1.34, 15.22 | 1.70 | .098 |
|  | Left PCL x Time x Tx Group | -4.38 | -18.14, 9.38 | -0.65 | .522 |
|  |  |  |  |  |  |
|  | Right SPL | -6.11 | -17.13, 4.90 | -1.12 | .270 |
|  | Time | 5.78 | 0.04, 11.52 | 2.05 | .048 |
|  | Tx Group | -3.35 | -13.42, 6.72 | -0.67 | .507 |
|  | Right SPL x Tx Group | 9.26 | -3.12, 21.63 | 1.50 | .139 |
|  | Right SPL x Time | 0.95 | -7.72, 9.62 | 0.22 | .825 |
|  | Time x Tx Group | 6.70 | -1.38, 14.78 | 1.69 | .101 |
|  | Right SPL x Time x Tx Group | -6.08 | -15.75, 3.58 | -1.28 | .210 |
|  |  |  |  |  |  |
|  | Left LG | 0.04 | -8.47, 8.54 | 0.09 | .993 |
|  | Time | 6.33 | 0.05, 12.62 | 2.05 | .048 |
|  | Tx Group | -2.94 | -13.55, 7.68 | -0.56 | .581 |
|  | Left LG x Tx Group | 0.43 | -10.42, 11.27 | 0.08 | .937 |
|  | Left LG x Time | -1.61 | -8.22, 5.00 | -0.50 | .623 |
|  | Time x Tx Group | 5.42 | -3.40, 14.23 | 1.25 | .220 |
|  | Left LG x Time x Tx Group | -1.05 | -9.52, 7.41 | -0.25 | .802 |

Note. Predictors were tested within a model that included a random intercept and slope. Time was a 2-level within-subjects variable that was coded such that 0 = baseline and 1 = post-treatment. Treatment group was a two-level between-subjects variable coded such that 0 corresponded to AP-AAT and 1 corresponded to Balanced AAT. Neural regions were areas whose activations to the push-positive relative to the pull-positive condition of the baseline AAT differed significantly between SAD patients and healthy controls. AP-AAT = Approach-positive approach-avoidance training; SCS-R = Social Connection Scale – Revised PCL = Paracentral lobule; SPL = Superior parietal lobule; LG = Lingual gyrus; Tx = Treatment; CI = Confidence interval.

Supplemental References

APA, A. P. A. (2000). *Diagnostic and statistical manual of mental disorders*. Washington, DC: American Psychiatric Association.

First, M. B., Gibbon, M,. (2004). The Structured Clinical Interview for DSM-IV Axis I Disorders (SCID-I) and the Structured Clinical Interview for DSM-IV Axis II Disorders (SCID-II). In M. J. Hilsenroth, Segal, D. M. (Ed.), *Comprehensive handbook of psychological assessment* (Vol. 2, pp. 134-143). Hoboken, NJ: John Wiley & Sons.

Goeleven, E., De Raedt, R., Leyman, L., & Verschuere, B. (2008). The Karolinska Directed Emotional Faces: A validation study. *Cognition & Emotion, 22*(6), 1094-1118. doi:10.1080/02699930701626582

Liebowitz, M. R. (1987). Social Phobia. In *Modern Problems of Pharmacopsychiatry* (Vol. 22, pp. 141-173): Karger.

Najmi, S., Kuckertz, J. M., & Amir, N. (2010). Automatic avoidance tendencies in individuals with contamination-related obsessive-compulsive symptoms. *Behav Res Ther, 48*(10), 1058-1062. doi:10.1016/j.brat.2010.06.007

Taylor, C. T., & Amir, N. (2012). Modifying automatic approach action tendencies in individuals with elevated social anxiety symptoms. *Behavior Research and Therapy, 50*(9), 529-536. doi:10.1016/j.brat.2012.05.004
